# Assessing Heart Rate Variability Using A 12 lead ECG In Patients With Alcohol Dependence Syndrome

**Published:** 2009-01-07

**Authors:** S Sucharita, Raynette Clark, Priya Sreedaran, K Srinivasan

**Affiliations:** 1 Core Physiological Laboratories, St John's Research Institute, Bangalore, India; 2 Department of Psychiatry, St John's Medical College and Hospital, Bangalore, India; 3 Alcorn State University, Alcorn State, Mississippi, United States of America

**Keywords:** 12 lead derived RR variability, heart rate variability, Alcohol dependence Syndrome

## Abstract

Heavy or hazardous drinking is associated with an increased risk of cardiac morbidity and mortality and this has been attributed to abnormalities in cardiac autonomic regulation. Current study aimed to assess the role of simple indices derived from 12 lead ECG in subjects with chronic alcohol dependence. Data suggested that alcohol group had significantly lower 12 lead ECG derived RR variability compared to age and gender matched controls. Study further supports the implication of 12 lead derived RR variability indices in various clinical settings.

Heavy or hazardous drinking is associated with an increased risk of cardiac morbidity and mortality and this has been attributed to abnormalities in cardiac autonomic regulation [1]. Studies have shown that abnormalities in cardiac autonomic regulation reverse with abstinence from drinking [2]. Various methods have been used to measure autonomic nervous system in harmful drinkers. Conventional clinical autonomic tests and of late heart rate variability are two of the most commonly used approaches to study cardiac autonomic regulation [3]. Lowered HRV is associated with increased risk of mortality and HRV has been proposed as a marker for disease [4]. However, measurement of HRV requires sophisticated computer processing and a well established laboratory. In a recent study we showed that RR variability can be measured from standard 12 lead ECG and found a good correlation between this measure and software derived RR variability in healthy human subjects of various age range [5]. However, it is not known whether RR variability derived from standard 12 lead ECG could detect abnormalities in autonomic regulation in clinical conditions such as alcohol abuse. Current study aimed to assess the cardiovascular autonomic responses in subjects with alcohol dependence syndrome using standard 12 lead ECG derived RR variability and compared it with age and gender matched healthy controls.

34 subjects were recruited including 17 with alcohol abuse (M:F, 16:1) and 17 age and gender matched controls. All subjects underwent a detailed clinical examination and relevant laboratory investigations. None of the subjects had any co-morbid medical conditions including any clinically significant autonomic symptoms. Alcohol Use Disorders Identification Test (AUDIT) [6] was used to assess the severity of alcohol abuse. A score of 8 or above indicates problem drinking. A standard 12 lead EGG (Cardiart 108T/MK-VII, BPL limited, India) was done on the second day of abstaining from alcohol. The RR intervals were calculated manually. At least 3 RR intervals were used in each lead to calculate Mean- RR, Standard deviation (SD)-RR, and Coefficient of variation (CV)-RR. In a subpopulation (n=12; all male) of alcohol dependence subjects 12 lead RR variability indices were also compared with the indices of power spectral analysis heart rate variability. The details of procedure and units of measurement have been discussed in our previous publication [3,5]. All participants gave written consent to the studies which were approved by the Institutional Ethics Review Board.

Subjects with alcohol abuse had an AUDIT score of 28.1±7.0 and healthy controls 1.05±1.43. There was a trend of positive association between CV-RR and LF absolute units (r=0.57, p=0.06); Total power (r=0.50, p=0.138) and HF in absolute units (r=0.16, p=0.63). Table 1 shows measures of RR variability derived from 12 lead ECG in subjects with alcohol abuse with their age and gender matched controls. Alcohol group had a significant decrement in all the 12 lead derived measures of RR variability compared to their age and gender matched controls (all, P<0.05). Heart rate was significantly higher in the alcohol group compared to the controls.

Data indicates that the alcohol group had lesser heart rate variability compared to their age and gender matched controls, suggesting involvement of cardiovascular autonomic nervous system. Results from the present study indicate that the use of standard 12 lead ECG for estimating RR variability could be a simple and cost effective method that could be employed by researchers and clinicians to assess cardiac autonomic regulation. Further, there is a need to explore RR variability derived from 12 lead ECG in prospective epidemiological studies linked to all cause and cardiac mortality.

## Figures and Tables

**Table 1:**
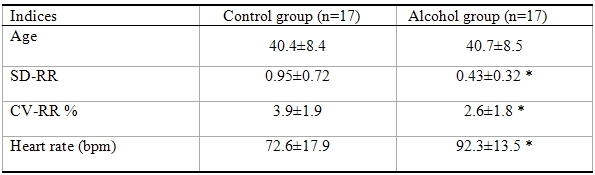
Demonstrating the 12 lead derived RR variability in alcohol and control groups

CV-RR, Coefficient of variation RR interval; SD-RR, Standard deviation RR interval * P<0.05
